# Supplementary material for: The gut hormone Allatostatin C/Somatostatin regulates food intake and metabolic homeostasis under nutrient stress
Source: Nat Commun. 2022 Feb 4;13:692. doi: 10.1038/s41467-022-28268-x (PMC8816919; doi:10.1038/s41467-022-28268-x)
Supplement: Supplementary file 1 — Supplementary Information [file 41467_2022_28268_MOESM1_ESM.pdf]

Supplemental information

The gut hormone Allatostatin C/Somatostatin regulates food intake and metabolic homeostasis under nutrient stress

Olga Kubrak[1,†], Takashi Koyama[1†], Nadja Ahrentløv[1,†], Line Jensen[1], Alina Malita[1], Muhammad T. Naseem[1], Mette Lassen[1], Stanislav Nagy[1], Michael J. Texada[1], Kenneth V. Halberg[1], and Kim Rewitz[1,]*

[†] Equal contribution

[1] Department of Biology, University of Copenhagen, 2100 Copenhagen O, Denmark

*Correspondence: Kim.Rewitz@bio.ku.dk

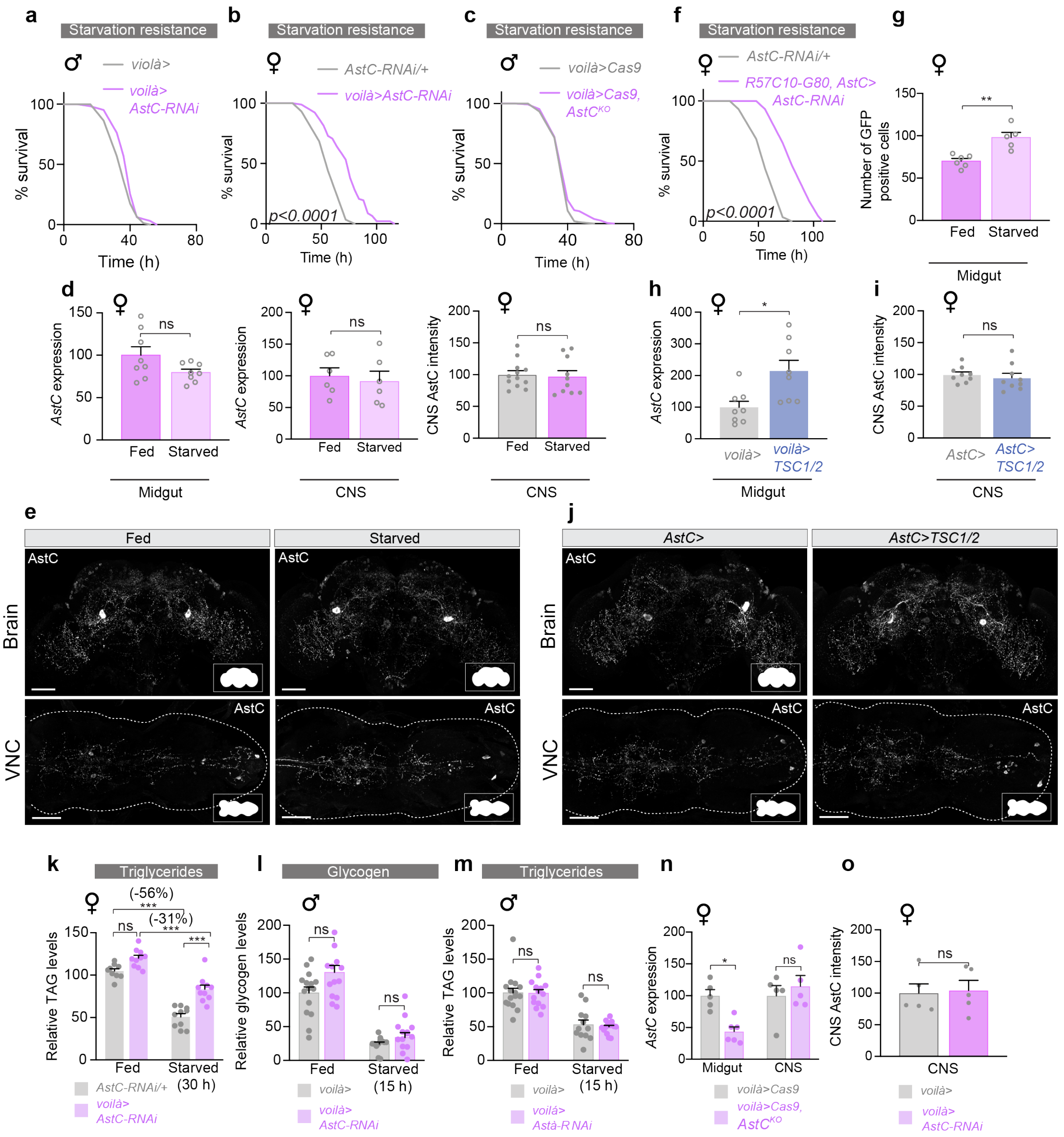

Figure S1. Gut-derived AstC does not alter starvation survival or starvation-induced energy mobilization in males; *AstC* is regulated by TOR signaling in females. a and **c**, Male flies with *AstC*-targeting RNAi **(a)** or CRISPR knockout **(c)** in their EECs restricted to the adult stage do not exhibit longer survival during starvation than controls. **b** and **f**, Adult females with *voilà>* driven **(b)** or *R57C10-G80*, *AstC>* driven **(f)** *AstC* knockdown exhibit increased survival during starvation compared to *AstC-RNAi/+* animals carrying only the RNAi construct without the GAL4 driver, indicating effects inherent to the RNAi line are not responsible for the observed increase in starvation resistance (p<0.0001 by Kaplan-Meier log-rank test). Data were obtained from different cohorts of animals. **d** and **e**, *AstC* transcript levels in the female central nervous system (CNS) and midgut, and CNS (brain and ventral nerve cord) AstC staining are not altered by 6-hour starvation (n=8 midgut and n=6 CNS for *AstC* transcripts; n=12 fed and n=10 starved for AstC immunostaining intensity). Scale bars 50 µm. **g**, The number of *AstC-GAL4*-expressing cells exhibiting observable *CaLexA>GFP* in the female gut is increased by starvation (p=0.0020; n=6 fed and n=5 starved guts). **h-j**, Inhibition of TOR via expressing *TSC1* and *TSC2* using *voilà>* in adult female for six hours (through temperature induction) increases *AstC* transcript levels in dissected female midguts (**h;** p=0.011), while six hours induction of TCS1/2 expression using *AstC>* does not alter CNS (brain and ventral nerve cord) AstC staining (**i** and **j**). In **h**, n=8; in **I,** n=9). Scale bars 50 µm. **k**, Females with EEC-specific *AstC* knockdown in the adult stage

(*voilà>AstC-RNAi*) carry increased TAG levels and they mobilize it more slowly compared to animals carrying the RNAi construct alone (*AstC-RNAi/+*), indicating that the RNAi line does not contribute to these effects (column 1 vs. 3, *p*<0.0001; column 2 vs. 4, *p*<0.0001; column 3 vs. 4, *p*<0.0001; two-way ANOVA genotype/diet interaction, *p*<0.01, all n=10 except n=9 for fed *voilà>*). Data were obtained from different cohorts of animals. **l** and **m,** *AstC* RNAi in the EECs does not alter the mobilization of glycogen **(l)** or TAGs **(m)** in males. In **l**, n=15 fed *voilà>*, n=13 fed *voilà>AstC-RNAi*, n=12 starved *voilà>*, n=14 starved *voilà>AstC-RNAi*; in **j,** n=15 fed *voilà>*, n=14 fed *voilà>AstC-RNAi*, n=12 starved *voilà>*, n=1 starved *voilà>AstC-RNAi*). **n**, CRISPR-mediated knockout of *AstC* in the EECs with *voilà>* reduces *AstC* transcripts in midguts, but not the CNS, indicating that the CRISPR efficiently disrupts the *AstC* locus and that the *voilà>* driver specifically targets gut and not brain *AstC* (*p*=0.010; all n=5 except n=6 for *voilà>Cas9,* $AstC^{KO}$ midgut). **o,** Quantification of immunostaining of AstC intensity in the CNS (brain and ventral nerve cord), related to images shown in Fig. 2F, indicates that *voilà>AstC-RNAi* does not affect AstC in the CNS (n=5). Error bars indicate standard error of the mean (SEM). ns, non-significant; *, *p*<0.05; and **, *p*<0.01. Statistical significance was determined using Kaplan-Meier log-rank tests (**b** and **f**), two-way ANOVA with Bonferroni's post hoc test (**k**, **l**, **m**, and **n**), and two-tailed Student's t-test (**d**, **g**, **h**, **i** and **o**). Source data are provided as a Source Data file.

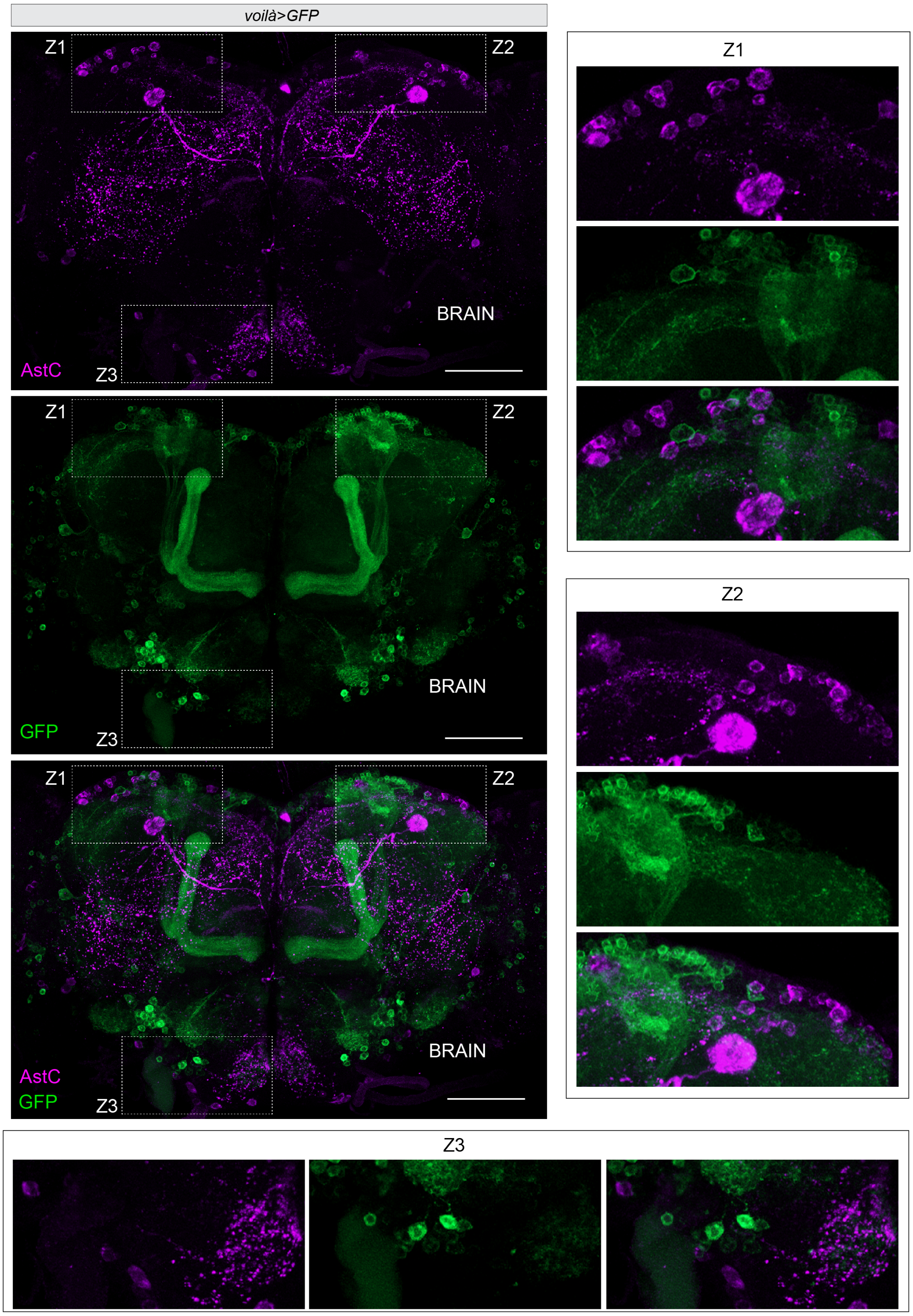

Figure S2. The *voilà>* driver that efficiently knocks *AstC* down in the gut does not target brain AstC. Immunostaining of the brain of an *voilà>GFP* female shows that GFP expression (green) does not overlap with neurons stained for AstC (magenta), indicating that although *voilà>* exhibits some neuronal expression it does not drive expression in AstC-positive cells, consistent with the results showing that RNAi or CRISPR against *AstC* driven using *voilà>* does not affect AstC levels in the nervous system. Zoom boxes Z1-3 show areas that are enlarged to clearly show the lack of overlap between GFP and AstC in certain brain regions. Images are representative of 8 scanned brains. Scale bars 50 µm.

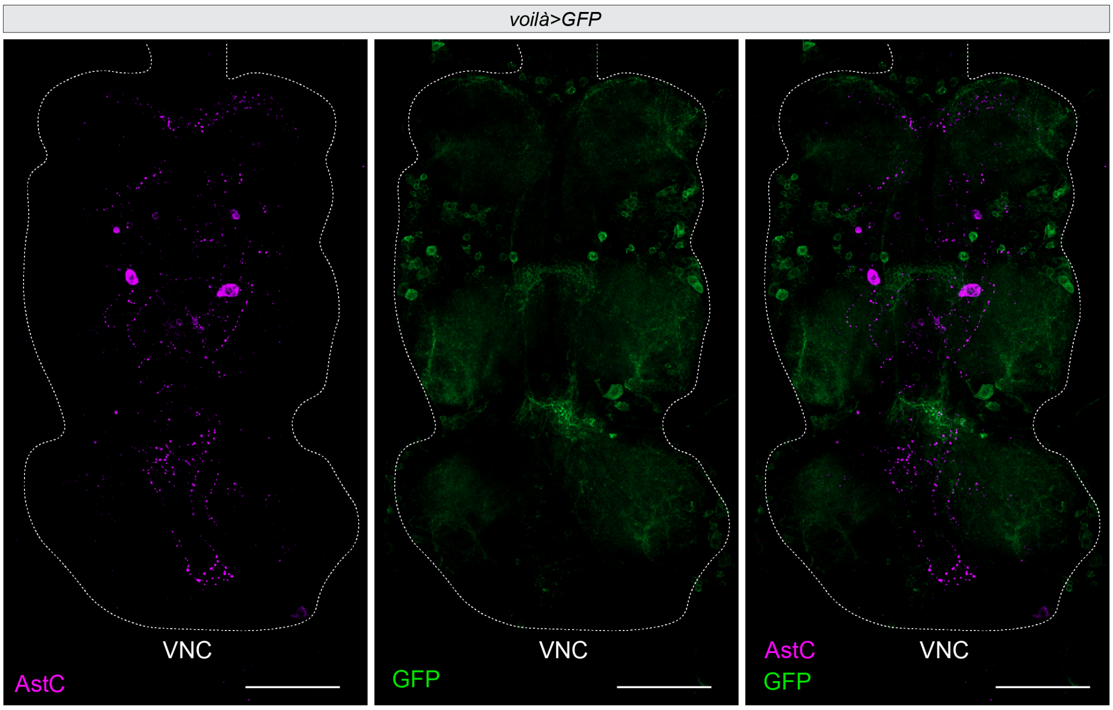

Figure S3. The *voilà>* driver that efficiently knocks *AstC* down in the gut does not target AstC in the ventral nerve cord. Immunostaining of the ventral nerve cord (VNC) from an *voilà>GFP* female shows that GFP staining (green) does not overlap with neuronal staining of AstC (magenta), indicating that *voilà>* does not drive expression in AstC-positive cells in the VNC. Images are representative of 8 scanned VNCs. Scale bars 50 µm.

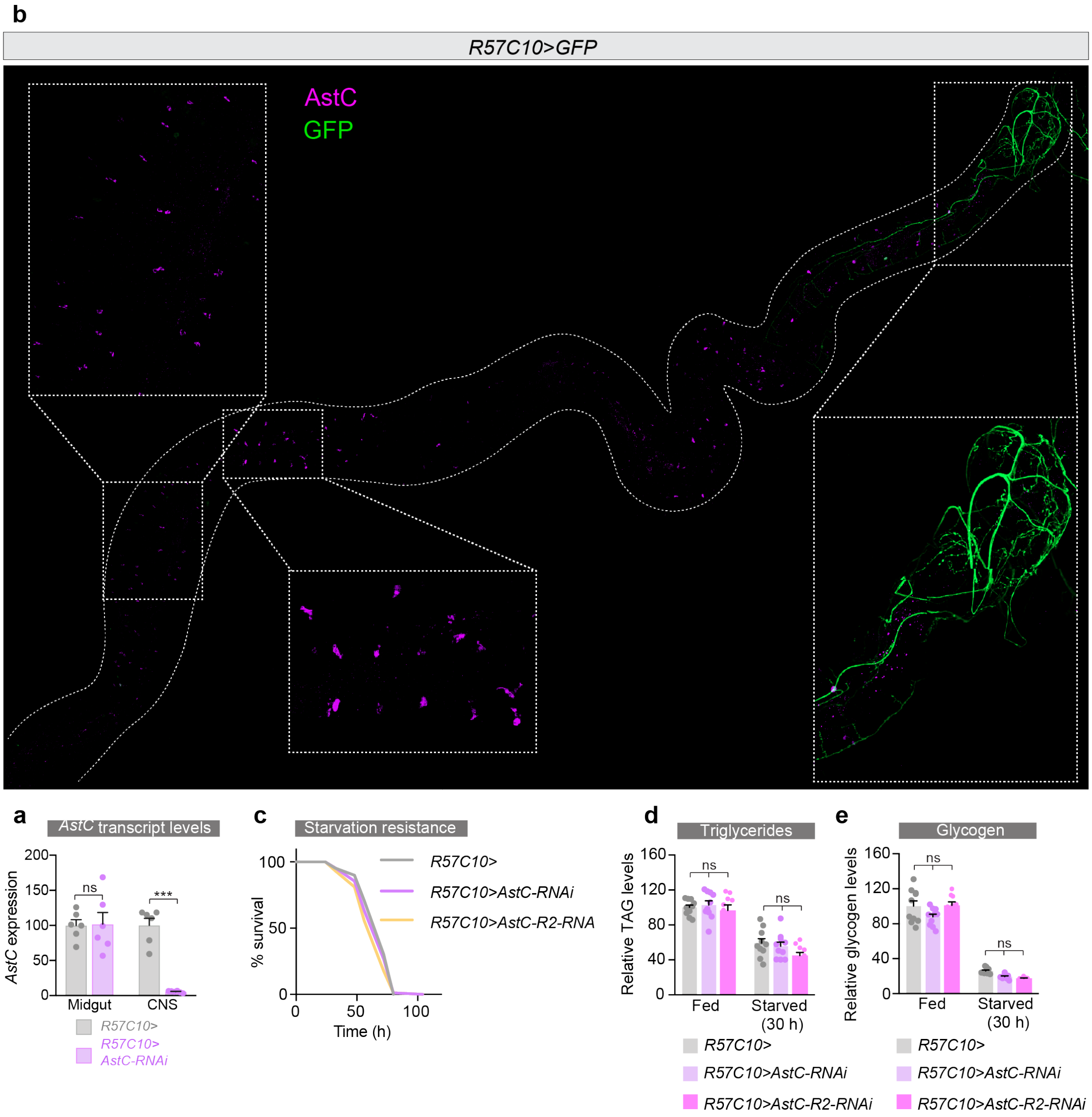

Figure S4. The pan-neuronal *R57C10>* driver (an *nSyb>* variant) reduces *AstC* in the nervous system, which does not increase starvation resistance or reduce starvation-induced energy mobilization. **a**, Pan-neuronal *AstC* knockdown using *R57C10>* strongly reduces *AstC* transcripts in the central nervous system (p<0.0001; CNS; brain and ventral nerve cord) without affecting expression in the midgut in adult females (n=6). **b**, Immunostaining of an adult female midgut from an *R57C10>GFP* animal shows GFP staining (green) in neuronal processes innervating the posterior midgut but not in AstC-positive cells (magenta) in the R3 middle midgut, consistent with results showing that this driver does not target AstC in the gut, but only the nervous system. Inset zooms clearly show that AstC and GFP stains do not overlap. **c-e**, Neuronal knockdown of *AstC* or its receptor *AstC-R2* does not increase starvation resistance **(c)** or lead to slower mobilization of TAG and glycogen during starvation **(d** and **e)**. in **d**, n=10; in **e** n=10 fed *R57C10>*, fed *R57C10>AstC-RNAi*, fed *R57C10>AstC-R2-RNAi*, starved *R57C10>AstC-RNAi*, starved *R57C10>AstC-R2-RNAi*, n=9 in starved *R57C10>*. Error bars indicate standard error of the mean (SEM). ns, non-significant; ***, p<0.001. Statistical significance was determined using Kaplan-Meier log-rank tests (**c**), two-way ANOVA with Bonferroni's post hoc test (**d** and **e**), and two-tailed Mann-Whitney U test (**a**). Source data are provided as a Source Data file.

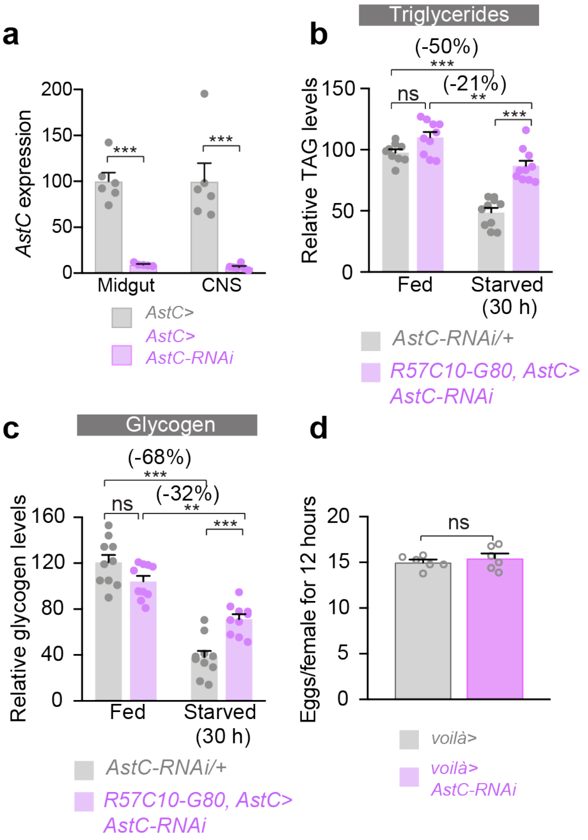

Figure S5. ***AstC*****> targets both nervous system and gut; loss of gut *AstC* does not influence fecundity; and the effects of AstC on metabolism are not caused by inherent RNAi line effects. a**, *AstC>AstC-RNAi* strongly knocks *AstC* down in both central nervous system (CNS; brain and ventral nerve cord) and midguts of adult females (column 1 vs. 2, p<0.0001; column 3 vs. 4, p<0.0001; all n=6 except n=5 for *AstC>AstC-RNAi* midgut). **b** and **c**, Animals with RNAi driven against *AstC* in the EECs deplete energy more slowly than the RNAi lines, indicating that effects are not due to inherent RNAi line effects. Data were obtained from different cohorts of animals. (In **b**, column 1 vs. 3, p<0.0001; column 2 vs. 4, p=0.0060; column 3 vs. 4, p<0.0001; all n=10 except n=9 for fed *AstC-RNAi/+*; in **c**, column 1 vs. 3, p<0.0001; column 2 vs. 4, p=0.0010; column 3 vs. 4, p=0.0005; all n=10). **d**, Egg-laying performance over 12 hours does not differ between controls (*voilà>*) and animals with knockdown of *AstC* in the EECs (*voilà>AstC-RNAi*). n=6. Error bars indicate standard error of the mean (SEM). ns, non-significant; **, p<0.01; and ***, p<0.001. Statistical significance was determined using two-way ANOVA with Bonferroni's post hoc test (**b** and **c**), two-tailed Mann-Whitney U test (**a**), and two-tailed Student's t-test (**d**). Source data are provided as a Source Data file.

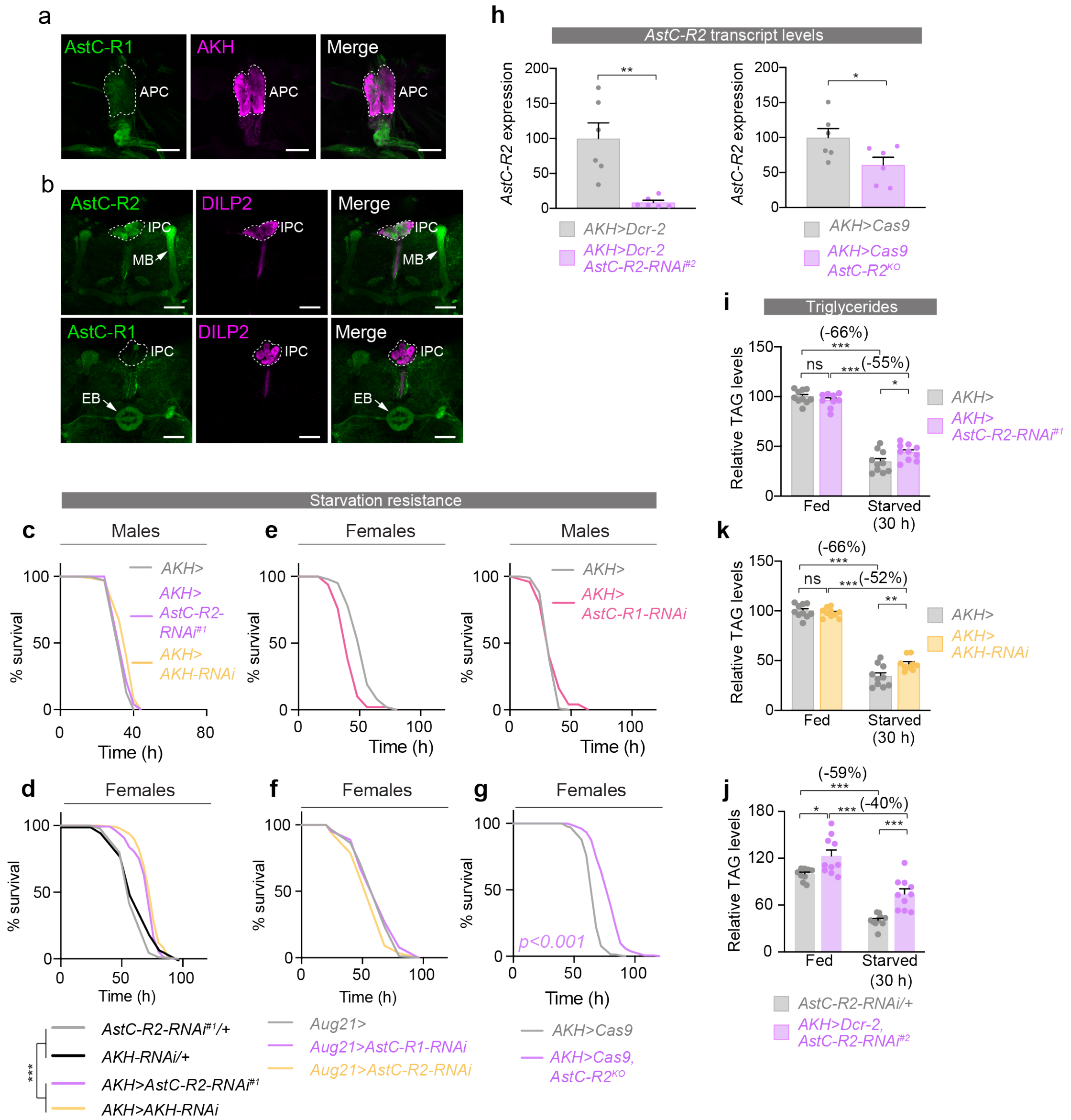

Figure S6. AstC effects are mediated by AstC-R2 in the APCs. a, *AstC-R1::2A::GAL4 > UAS-GFP* (green) does not strongly mark the female CC (marked by anti-AKH, magenta). Images are representative of 8 images. Scale bars 20 µm. **b**, *AstC-R2::2A::GAL4>GFP* (green) reports AstC-R2 expression in the IPCs (marked by anti-DILP2, magenta) and the mushroom bodies ("MB"); *AstC-R1>GFP* reports AstC-R1 expression in the ellipsoid body ("EB") of the central complex of the brain, but not in the IPCs (anti-DILP2, magenta). Images are representative of five brains scanned. Scale bars 25 µm. **c**, Knockdown of *AstC-R2* in the APCs does not prolong male starvation survival. Knockdown of *AKH* itself in the male APCs also has no effect on starvation survival. **d**, Knockdown of *AstC-R2* or *AKH* in the APCs of adult females prolongs starvation resistance compared to RNAi lines without GAL4 driver (p<0.0001 by Kaplan-Meier log-rank test). Data were obtained from different cohorts of animals. **e**, Knockdown of *AstC-R1* in the APCs does not prolong starvation in survival in females (left panel) or males (right panel), compared with controls. **f**, Knockdown of *AstC-R1* or *AstC-R2* in the corpora allata (CA), which produce juvenile hormone (JH), using the CA-specific *Aug21-GAL4* with GAL80^{TS} (*Aug21>*) does not prolong starvation survival in females, indicating that effects of AstC are not mediated by effects on JH. **g**, CRISPR-mediated deletion of *AstC-R2* in the female APCs phenocopies the starvation-survival effect of *AstC-R2-RNAi* in this tissue. **h**, RNAi-mediated knockdown or CRISPR-mediated knockout of *AstC-R2* in the APCs with *AKH>* reduces *AstC* transcripts in the APCs, indicating that the RNAi and CRISPR efficiently disrupt *AstC* expression (left panel, p=0.0022; right panel, p=0.044; n=6). **(i-k)** Adult-restricted RNAi against *AstC-R2* (**i**) or *AKH* (**k**) in the female APCs has similar suppressive effects on TAG mobilization during 30-hour starvation (In **i**, column 1 vs. 3, p<0.0001; column 2 vs. 4, p<0.0001;

column 3 vs. 4, p=0.029; In **k**, column 1 vs. 3, p<0.0001; column 2 vs. 4, p<0.0001; column 3 vs. 4, p=0.0069; two-way ANOVA genotype/diet interaction, p<0.05, all n=10 except n=9 for fed *AKH>AstC-R2-RNAi* and n=9 fed *AKH>AKH-RNAi).* **j**, This effect is not due to the *AstC-R2* RNAi line itself (column 1 vs. 2, p=0.019; column 1 vs. 3, p<0.0001; column 2 vs. 4, p<0.0001; column 3 vs. 4, p=0.0003; two-way ANOVA genotype/diet interaction, p<0.01, all n=10). Data were obtained from different cohorts of animals in **j**. Error bars indicate standard error of the mean (SEM). ns, non-significant; *, p<0.05; **, p<0.01; and ***, p<0.001. Statistical significance was determined using Kaplan-Meier log-rank tests (**c**, **d**, **e**, **f**, and **g**), two-way ANOVA with Bonferroni's post hoc test (**i**, **j**, and **k**), and two-tailed Mann-Whitney U test and and two-tailed Student's t-test (**h**). Source data are provided as a Source Data file.

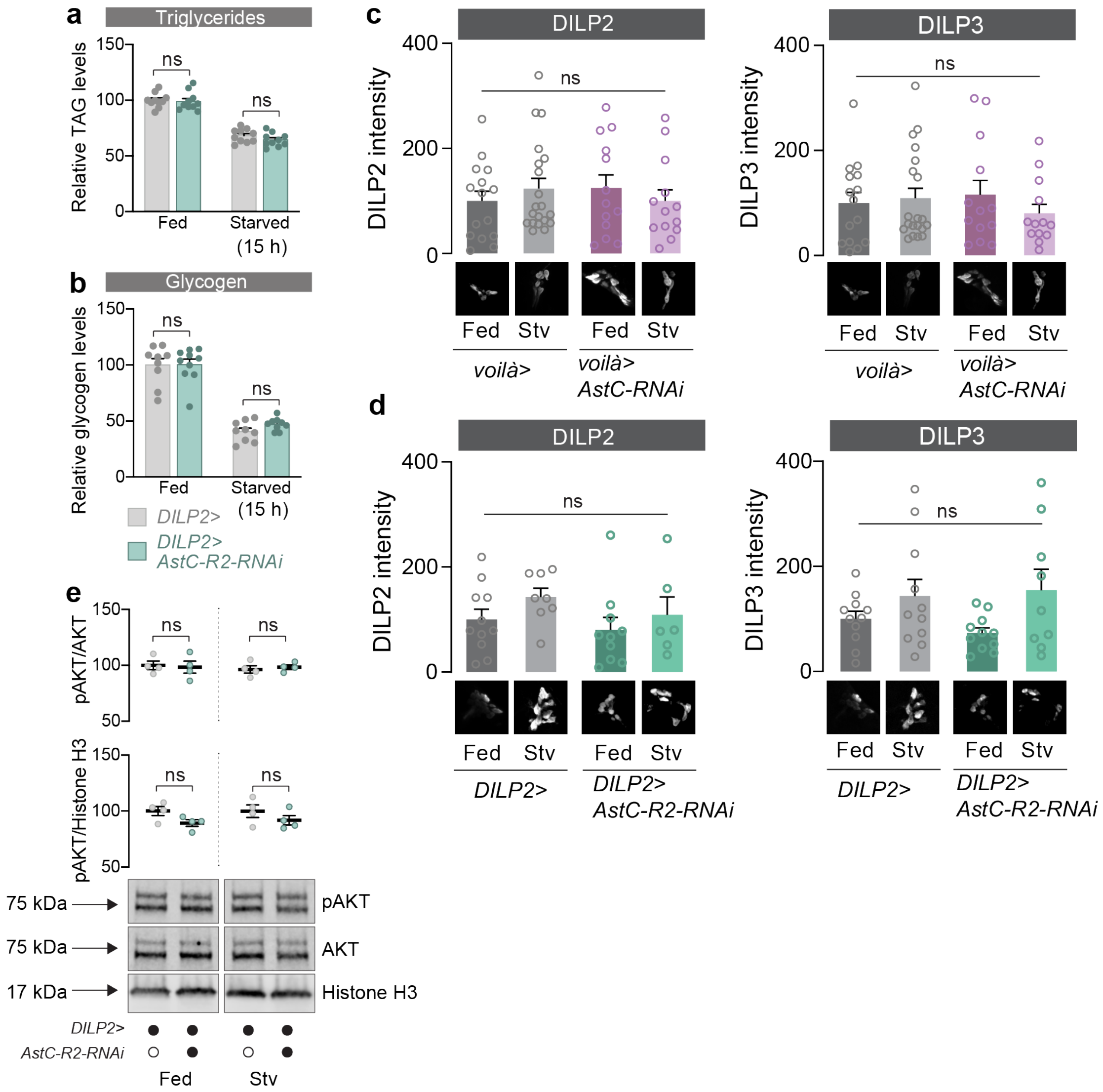

Figure S7. Although AstC-R2 appears to be expressed in the IPCs, it does not significantly affect insulin signaling or energy mobilization under the nutrient-stress conditions examined. a and **b**, Knockdown of *AstC-R2* in the IPCs does not alter the mobilization of glycogen **(a)** or TAG **(b)** during 15-hour starvation. In **a**, n=10; in **b**, n=9 except n=10 fed *DILP2>AstC-R2-RNAi*. **c**, Knockdown of *AstC* in the EECs does not alter the accumulation of DILP2 (left panel) or DILP3 (right panel) in female IPCs, as measured by anti-DILP immunostaining (n=15 fed *voilà>*, n=13 fed *voilà>AstC-RNAi*, n=20 starved *voilà>*, and n=13 fed *voilà>AstC-RNAi*). **d**, Likewise, knockdown of *AstC-R2* in the IPCs does not strongly alter the accumulation of DILP2 (left) or DILP3 (right) in these cells (DILP2 intensity, n=11 fed *DILP2>*, n=10 fed *DILP2>AstC-R2-RNAi*, n=8 starved *DILP2>*, n=6 starved *DILP2>AstC-R2-RNAi*; DILP3 intensity, n=11 fed *DILP2>*, fed *DILP2>AstC-R2-RNAi*, starved *DILP2>*, and n=9 starved *DILP2>AstC-R2-RNAi*). **e**, Knockdown of *AstC-R2* in the IPCs does not strongly alter peripheral insulin-signaling activity during feeding or starvation, as measured by anti-phospho-AKT Western blotting of whole females. Top: anti-phospho-AKT (pAKT) staining levels normalized against total AKT staining; middle: anti-pAKT staining normalized against histone H3 staining; bottom: Western-blot lanes illustrating similar staining intensities under all conditions (n=4). The full blots, including mass markers, are appended to this file. Error bars indicate standard error of the mean (SEM). ns, non-significant. Statistical significance was determined using two-way ANOVA with Bonferroni's post hoc test (**a**, **b**, **c**, and **d**) and two-tailed Student's t-test (**e**). Source data are provided as a Source Data file.

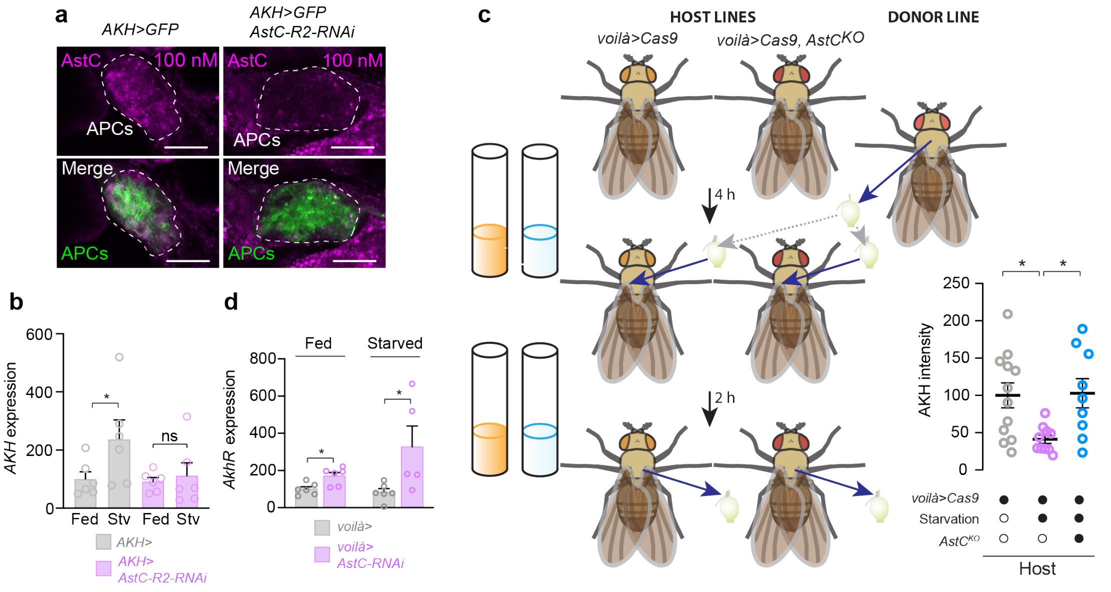

Figure S8. APCs bind AstC in an AstC-R2-dependent manner; transcript levels of *AKH* and *AkhR* are altered with disruption of AstC signaling; transplantation scheme. a, Fluorescently labeled AstC peptide (magenta) binds to the APCs (marked by *AKH>GFP*, green) of normal animals, whereas staining is much weaker in APCs expressing *AstC-R2-RNAi*. Images are representative of two preparations. Scale bar, 25 µm. **b**, In control animals, *AKH* transcript levels are increased with starvation (*p*=0.041); this upregulation does not occur, however, when *AstC-R2* is knocked down in the APCs (n=6). **c**, Transplantation scheme: Adult female APCs (CCs) were transplanted into adult female control hosts (*voilà>Cas9*) or hosts with EEC-specific *AstC* knockout (*voilà>Cas9, $AstC^{KO}$*) that were either fed or pre-starved for 4 hours. After 2 hours' incubation within the host animals, the CCs were recovered and stained for AKH. Incubation within a fasting host induces AKH release, but not if *AstC* is deleted in the host EECs (column 1 vs. 2, *p*=0.040; column 2 vs. 3, *p*=0.034; n=12 *voilà>Cas9*, n=10 *voilà>Cas9* starvation, n=9 *voilà>Cas9, $AstC^{KO}$* starvation). **d**, Expression of *AkhR* is higher in animals in which EEC expression of *AstC* is knocked down (*p*=0.017; n=6 fed *voilà>*, fed *voilà>AstC-RNAi*, starved *voilà>*, and n=5 starved *voilà>AstC-RNAi*). Error bars indicate standard error of the mean (SEM). ns, non-significant. *, *p*<0.05. Statistical significance was determined using one-way ANOVA with Kruskal-Wallis test (**c**) and two-tailed Mann-Whitney U test (**b** and **d**). Source data are provided as a Source Data file.

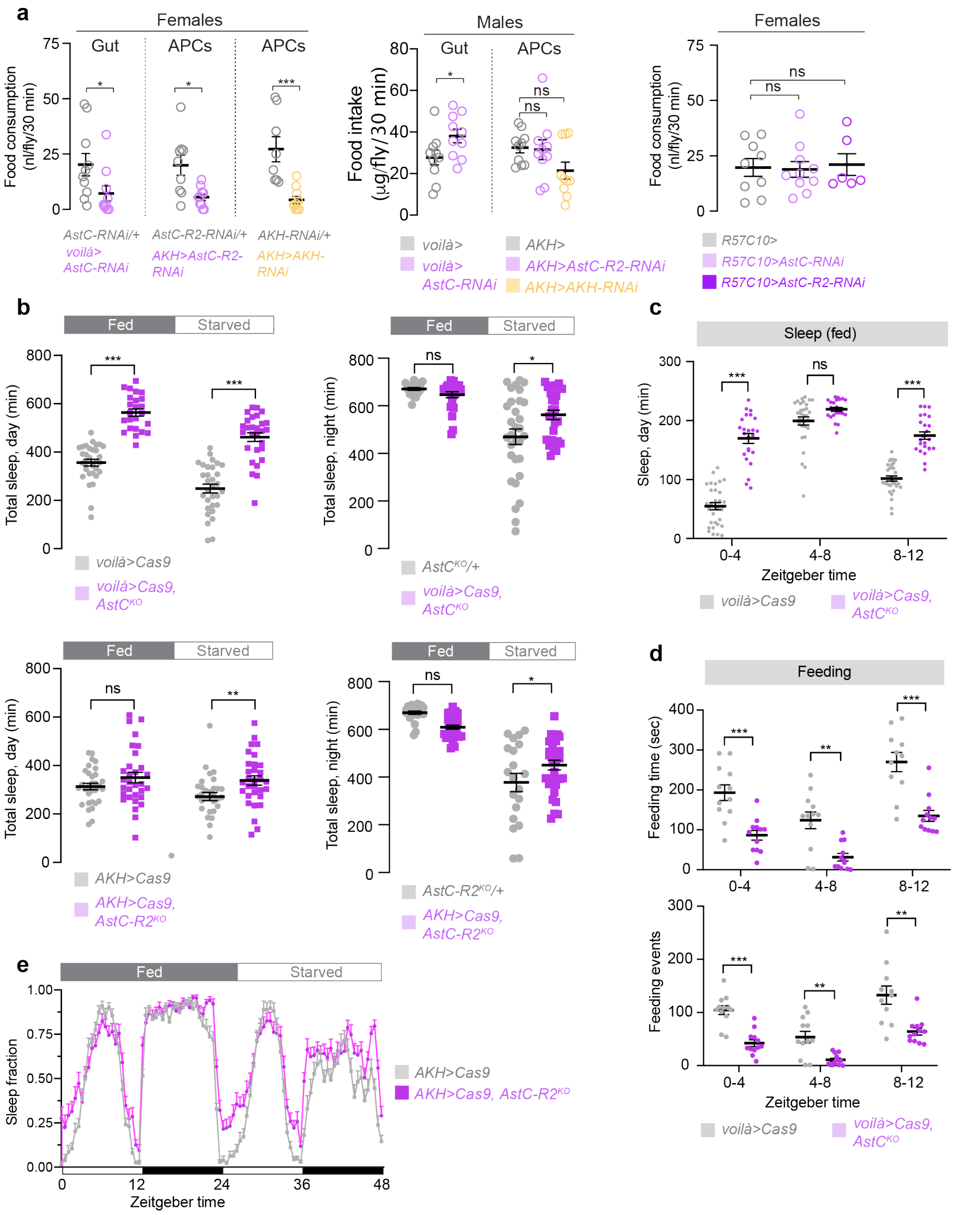

Figure S9. Effects of AstC and AKH signaling on feeding in males and on feeding and sleep in females. a, Food intake over 30 minutes, measured by a dye-feeding assay, shows that the reduced food intake of females with *AstC* knockdown in the EECs or of *AstC-R2* or *AKH* in the APCs does not arise from the RNAi line alone (left panel, gut, p=0.023; APCs middle, p=0.015; APCs right, p=0.0003; n=10 except n=8 *AstC-R2-RNAi/+* and n=8 *AKH-RNAi/+*); furthermore, this reduced intake is not observed in males (middle panel, gut, p=0.049; n=10) or in females with neuronal knockdown of *AstC* or *AstC-R2* (right panel, n=9 *R57C10>*, n=10 *R57C10>AstC-RNAi*, n=6 *R57C10>AstC-R2-RNAi*). **b**, *AstC* knockout in the EECs or *AstC-R2* knockout in the APCs increases the amount of daytime sleep under fed and starved conditions (left panels); the RNAi and guide-RNAi constructs alone induce different effects (left top panel, column 1 vs. 2, p<0.0001; column 3 vs. 4, p<0.0001; right top panel, column 3 vs. 4, p=0.021; left bottom panel, column 3 vs. 4, p=0.0047; right bottom panel, column 3 vs. 4, p=0.032; n=31 in fed and starved *AKH>Cas9* and *AKH>Cas9,AstC-R2^{KO}*; n=31 in fed and starved *voilà>Cas9*, n=24 fed *voilà>Cas9, AstC^{KO}*, n=28 starved *voilà>Cas9, AstC^{KO}*; n=20 fed and n=31 starved *AstC^{KO}/+*; n=23 fed

and n=20 starved *AstC-R2*^{KO}/+). **c** and **d**, Female daytime sleep **(c)** and feeding **(d)** binned into four-hour periods shows that *voilà>Cas9, AstC*^{KO} animals exhibit reduced feeding behavior during the middle of the day (Zeitgeber time 4-8), although their sleep amount is similar to controls' (*voilà>Cas9*), indicating that reduced feeding is not simply a consequence of increased sleep. In **c**, column 1 vs. 2, $p<0.0001$; column 4 vs. 5, $p<0.0001$; n=31 *voilà>Cas9*, n=24 *voilà>Cas9, AstC*^{KO}; in **d**, top panel column 1 vs. 2, $p=0.0002$; column 1 vs. 2, $p=0.0037$; column 4 vs. 5, $p=0.0002$; bottom panel column 1 vs. 2, $p<0.0001$; column 1 vs. 2, $p=0.0046$; column 4 vs. 5, $p=0.0005$; n=12 Zeitgeber time 0-4 and 8-12, n=11 Zeitgeber time 4-8 *voilà>Cas9*, n=12 Zeitgeber time 4-8 *voilà>Cas9, AstC*^{KO}. **e**, Sleep in *AKH>Cas9* and *AKH>Cas9, AstC-R2*^{KO} during fed conditions (hours 0 through 24); during starvation (hours 24 through 48). n=31. Error bars indicate standard error of the mean (SEM). ns, non-significant; *, $p<0.05$. Statistical significance was determined using one-way ANOVA with Kruskal-Wallis test (**a**) and two-tailed Mann-Whitney U test or two-tailed Student's t-test (**b**, **c** and **d**). Source data are provided as a Source Data file.

Table S1. Transgenic RNAi lines used for screening.

Target gene	Gene name	VDRC Stock Number
Actβ	*Activin beta*	108663
Akh	*Adipokinetic hormone*	105063
amn	*amnesiac*	5606
AstC	*Allatostatin C*	102735
CCHa2	*CCHamide 2*	102257
CNMa	*CNMamide*	104599
daw	*dawdle*	105309
Dh44	*Diuretic hormone 44*	108473
gbb	*glass-bottom boat*	330684
Gbp1	*Growth-blocking peptide 1*	108755
Gbp2	*Growth-blocking peptide 2*	330018
Gbp3	*Growth-blocking peptide 3*	6838
grk	*gurken*	101701
hh	*hedgehog*	109454
ITP	*Ion Transport Peptide*	43848
Lst	*Limostatin*	106861
ntl	*natalisin*	19547
NPF	*Neuropeptide F*	330277
Nplp2	*Neuropeptide-like precursor 2*	15306
Nplp4	*Neuropeptide-like precursor 4*	104662
Orcokinin	*Orcokinin*	106882
Proc	*Proctolin*	102488
Pvf1	*PDGF- and VEGF-related factor 1*	102699
Pvf3	*PDGF- and VEGF-related factor 3*	105008
pyr	*pyramus*	36524
RYa	*Ryamide*	109267
scw	*screw*	105303
spi	*spitz*	103187
spz	*spätzle*	105017
spz3	*spätzle 3*	102871
Tk	*Tachykinin*	103662
upd3	*unpaired 3*	106869
vn	*vein*	109437
Wnt4	*Wnt oncogene analog 4*	104671

Table S2. Sequences of qPCR primers and gRNA target sites.

QPCR Oligo	Sequence
AstC-F	TACGGCCTACTCCTCACCC
AstC-R	GCTGGCATATCGTAGCCACC
AstC-R2-F	AAGGACACTCGACACCAAATG
AstC-R2-R	TGTCAGCACAAATAACAGGATGC
Akh-F	TCCCAAGAGCGAAGTCCTCA
Akh-R	CCAGAAAGAGCTGTGCCTGA
AkhR-F	GCAAAAGTAGCTGAGGAGAATGA
AkhR-R	ATCCTTGGTCAGGTGAATGGT
Rp49-F	AGTATCTGATGCCCAACATCG
Rp49-R	CAATCTCCTTGCGCTTCTTG
**CRISPR Target**	**Sequence**
AstC target #1	GTGCAGATATTATTGTGCTA
AstC target #2	GTTAGTTTAACCAAGAGCTC
AstC-R2 target #1	GATCAACAGGAAGATCGAGG
AstC-R2 target #2	GTTACCTGTGAAATCCAGTG

Uncropped Western blots related to supplementary figure 7e

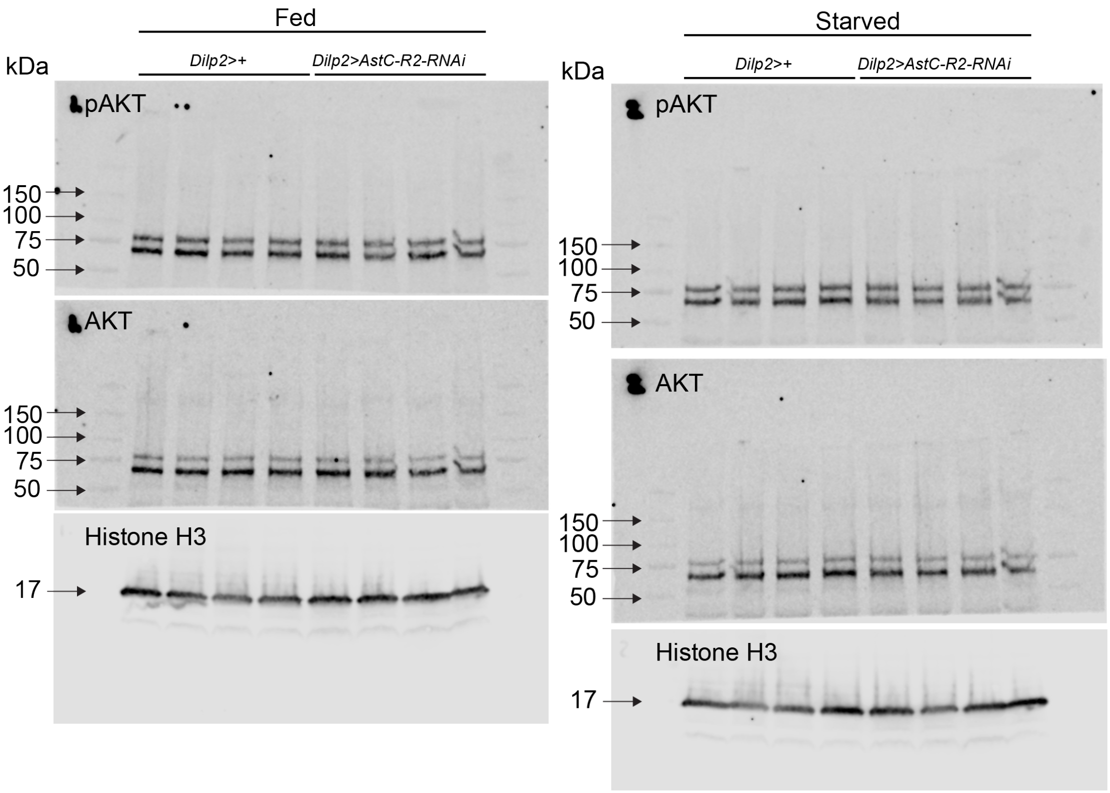

Note that all primary antibodies were raised in rabbits. The membranes were cut above the Histone H3 mass to permit separate staining of pAkt and H3 without stripping. Total Akt was stained after stripping the membranes.
