# Supplementary material for: The gut hormone Allatostatin C/Somatostatin regulates food intake and metabolic homeostasis under nutrient stress
Source: Nat Commun. 2022 Feb 4;13:692. doi: 10.1038/s41467-022-28268-x (PMC8816919; doi:10.1038/s41467-022-28268-x)
Supplement: Supplementary file 3 — Reporting Summary [file 41467_2022_28268_MOESM3_ESM.pdf]

nature research

Corresponding author(s): REWITZ, Kim

Last updated by author(s): Dec 12, 2021

Reporting Summary

Nature Research wishes to improve the reproducibility of the work that we publish. This form provides structure for consistency and transparency in reporting. For further information on Nature Research policies, see our Editorial Policies and the Editorial Policy Checklist.

Statistics

For all statistical analyses, confirm that the following items are present in the figure legend, table legend, main text, or Methods section.

n/a	Confirmed	
☐	☒	The exact sample size (n) for each experimental group/condition, given as a discrete number and unit of measurement
☒	☐	A statement on whether measurements were taken from distinct samples or whether the same sample was measured repeatedly
☐	☒	The statistical test(s) used AND whether they are one- or two-sided<br>*Only common tests should be described solely by name; describe more complex techniques in the Methods section.*
☒	☐	A description of all covariates tested
☐	☒	A description of any assumptions or corrections, such as tests of normality and adjustment for multiple comparisons
☐	☒	A full description of the statistical parameters including central tendency (e.g. means) or other basic estimates (e.g. regression coefficient) AND variation (e.g. standard deviation) or associated estimates of uncertainty (e.g. confidence intervals)
☐	☒	For null hypothesis testing, the test statistic (e.g. F, t, r) with confidence intervals, effect sizes, degrees of freedom and P value noted<br>*Give P values as exact values whenever suitable.*
☒	☐	For Bayesian analysis, information on the choice of priors and Markov chain Monte Carlo settings
☒	☐	For hierarchical and complex designs, identification of the appropriate level for tests and full reporting of outcomes
☒	☐	Estimates of effect sizes (e.g. Cohen's d, Pearson's r), indicating how they were calculated

Our web collection on statistics for biologists contains articles on many of the points above.

Software and code

Policy information about availability of computer code

Data collection	Image acquisition: Zeiss ZEN<br>Acquisition of sleep data through DAM system: DAMSystem3 v3.10.7 software (TriKinetics, Inc.); Acquisition of FLIC data: embedded software within FLIC master controller
Data analysis	Statistical analyses: GraphPad PRISM, version 9 or earlier (continually updated)<br>Image analysis: NIH FIJI version 1.53c or earlier (continually updated)<br>Analysis of sleep data: pySolo (v1.1; published previously and cited in paper) and custom scripts in MATLAB (The MathWorks), available at https://zenodo.org/record/5772445#.YbNYAb3MKUk Analysis of FLIC data: previously published R package, cited in paper

For manuscripts utilizing custom algorithms or software that are central to the research but not yet described in published literature, software must be made available to editors and reviewers. We strongly encourage code deposition in a communiy repository (e.g. GitHub). See the Nature Research guidelines for submitting code & software for further information.

Data

Policy information about availability of data

All manuscripts must include a data availability statement. This statement should provide the following information, where applicable:

- Accession codes, unique identifiers, or web links for publicly available datasets; A list of figures that have associated raw data
- A description of any restrictions on data availability

Source data is available in Source Data files. The authors are not aware of repositories for raw Drosophila sleep or feeding data (Figs. 5 and S9), and therefore these types of raw data can be obtained directly from the authors upon reasonable request.

Field-specific reporting

Please select the one below that is the best fit for your research. If you are not sure, read the appropriate sections before making your selection.

☒ Life sciences ☐ Behavioural & social sciences ☐ Ecological, evolutionary & environmental sciences

For a reference copy of the document with all sections, see nature.com/documents/nr-reporting-summary-flat.pdf

Life sciences study design

All studies must disclose on these points even when the disclosure is negative.

Sample size	No sample-size calculations were performed. The number of samples is large enough to capture normal variation while maintaining feasibility for preparation. Sample numbers meet or exceed standards in our field. qPCR used 6-8 samples, each containing several tissues or animals, the standard in our lab; starvation and metabolic assays used 10-20 replicates containing multiple animals. Image analyses made use of multiple tissues per genotype or condition, as described in the appropriate figure legends or methods.
Data exclusions	No data are excluded
Replication	Experimental results with RNAi were confirmed with CRISPR, eliminating almost all chance of off-target effects. Representative images were chosen from multiple options, generally at least 6. All experiments producing numerical data include at least 5 replicates. All attempts at replication were successful.
Randomization	Animals were randomly grouped into batches as indicated in the text
Blinding	Researchers were not blinded during the study because this is not generally done in fly studies -- in many cases, different genotypes are discernible by the naked eye anyway. With limited staff with expertise in these particular studies, the person handling sample prep must usually also be the one performing the assay.

Reporting for specific materials, systems and methods

We require information from authors about some types of materials, experimental systems and methods used in many studies. Here, indicate whether each material, system or method listed is relevant to your study. If you are not sure if a list item applies to your research, read the appropriate section before selecting a response.

Materials & experimental systems

n/a	Involved in the study	
☐	☒	Antibodies
☒	☐	Eukaryotic cell lines
☒	☐	Palaeontology and archaeology
☐	☒	Animals and other organisms
☒	☐	Human research participants
☒	☐	Clinical data
☒	☐	Dual use research of concern

Methods

n/a	Involved in the study	
☒	☐	ChIP-seq
☒	☐	Flow cytometry
☒	☐	MRI-based neuroimaging

Antibodies

Antibodies used

Academic antibodies:
Rabbit anti-Akh was a gift of Jae Park, U. Tennessee, used 1:500;
Mouse anti-Prospero #MR1A supernatant was obtained from University of Iowa Developmental Studies Hybridoma Bank), used 1:20;
Rabbit anti-DILP2 was a gift of Ernst Hafen (ETH Zurich), used 1:1000;
Mouse anti-DILP3, used 1:500, and rabbit anti-AstC, used 1:500, were gifts of Jan Veenstra, University of Bordeaux.
Commercial antibodies:
Rabbit anti-phospho-Akt: Cell Signaling Technology #4054, 1:1000;
Rabbit anti-total-Akt: Cell Signaling Technology #4691, 1:1000;
Mouse anti-histone H3: Abcam, #ab1791, 1:1000;
Mouse anti-GFP, ThermoFisher #A11120, 1:500;
Alexa Fluor 488-conjugated goat anti-mouse, ThermoFisher #A32723, 1:500;
Alexa Fluor 488-conjugated goat anti-rabbit, ThermoFisher #A11008, 1:500;
Alexa Fluor 555-conjugated goat anti-mouse, ThermoFisher #A32732, 1:500;
IRDye800CW-conjugated anti-rabbit, LI-COR #925-32210, 1:10k;
IRDye680RD-conjugated anti-mouse, LI-COR #925-68070, 1:10k.

Validation

anti-Akh: Lee, G. & Park, J. H. Hemolymph sugar homeostasis and starvation-induced hyperactivity affected by genetic manipulations of the adipokinetic hormone-encoding gene in Drosophila melanogaster. Genetics 167, 311-323, doi:10.1534/genetics.167.1.311 (2004). **anti-AstC:** Veenstra, J. A., Agricola, H. J. & Sellami, A. Regulatory peptides in fruit fly midgut. Cell Tissue Res 334, 499-516, doi:10.1007/s00441-008-0708-3 (2008). **anti-DILP2:** Bader, R. et al. The IGFBP7 homolog Imp-L2 promotes insulin signaling in distinct neurons of the Drosophila brain. J Cell Sci 126, 2571-2576, doi:10.1242/jcs.120261 (2013).

anti-DILP3: Veenstra, J. A., Agricola, H. J. & Sellami, A. Regulatory peptides in fruit fly midgut. Cell Tissue Res 334, 499-516, doi:10.1007/s00441-008-0708-3 (2008). **anti-Pros:** Campbell, G. et al. RK2, a glial-specific homeodomain protein required for embryonic nerve cord condensation and viability in Drosophila. Development 120, 2957-2966 (1994). **Cell Signaling Technologies anti-phospho-Akt and anti-Akt sales pages attest to reactivity in Drosophila, as does Abcam 1791 page:**

https://www.cellsignal.com/products/primary-antibodies/phospho-drosophila-akt-ser505-antibody/4054
https://www.cellsignal.com/products/primary-antibodies/akt-pan-c67e7-rabbit-mab/4691
https://www.abcam.com/histone-h3-antibody-nuclear-marker-and-chip-grade-ab1791.html

Animals and other organisms

Policy information about studies involving animals; ARRIVE guidelines recommended for reporting animal research

Laboratory animals	Transgenic Drosophila melanogaster, non-invasive, non-pest species- Adult animals were used, mostly females but some males as well.
Wild animals	none
Field-collected samples	none
Ethics oversight	none required

Note that full information on the approval of the study protocol must also be provided in the manuscript.
